# Genetic Insights into Biofilm Formation by a Pathogenic Strain of *Vibrio harveyi*

**DOI:** 10.3390/microorganisms12010186

**Published:** 2024-01-17

**Authors:** Amandine Morot, François Delavat, Alexis Bazire, Christine Paillard, Alain Dufour, Sophie Rodrigues

**Affiliations:** 1 Laboratoire de Biotechnologie et Chimie Marines, Université Bretagne Sud, EMR CNRS 6076, IUEM, 56100 Lorient, France; 2 Univ Brest, CNRS, IRD, Ifremer, LEMAR, F-29280 Plouzané, France; 3 Nantes Université, CNRS, US2B, UMR6286, Nantes, France

**Keywords:** *Vibrio harveyi*, biofilm, transcriptome, type III secretion system, Tad pilus

## Abstract

The *Vibrio* genus includes bacteria widely distributed in aquatic habitats and the infections caused by these bacteria can affect a wide range of hosts. They are able to adhere to numerous surfaces, which can result in biofilm formation that helps maintain them in the environment. The involvement of the biofilm lifestyle in the virulence of *Vibrio* pathogens of aquatic organisms remains to be investigated. *Vibrio harveyi* ORM4 is a pathogen responsible for an outbreak in European abalone *Haliotis tuberculata* populations. In the present study, we used a dynamic biofilm culture technique coupled with laser scanning microscopy to characterize the biofilm formed by *V. harveyi* ORM4. We furthermore used RNA-seq analysis to examine the global changes in gene expression in biofilm cells compared to planktonic bacteria, and to identify biofilm- and virulence-related genes showing altered expression. A total of 1565 genes were differentially expressed, including genes associated with motility, polysaccharide synthesis, and quorum sensing. The up-regulation of 18 genes associated with the synthesis of the type III secretion system suggests that this virulence factor is induced in *V. harveyi* ORM4 biofilms, providing indirect evidence of a relationship between biofilm and virulence.

## 1. Introduction

Bacteria belonging to the *Vibrio* genus are ubiquitous in aquatic environments, being found in estuaries, marine coastal waters and sediments, and aquaculture settings. Some of these bacteria can form symbiotic or pathogenic relationships with a wide range of hosts. Thus, they alternate between survival and growth in aquatic environments and hosts [1]. One of the strategies adopted by *Vibrio* species to persist in changing environments is the conversion to a viable but non-culturable state (VBNC) [2]. Another strategy is the formation of biofilm which increases resistance to various stresses [3,4].

Biofilms are communities of microorganisms, usually attached to a surface, embedded in a self-produced extracellular matrix mainly composed of extracellular polysaccharides, DNA, and proteins. Changes in gene expression during biofilm formation allow the production of molecules and structures responsible for adhesion, aggregation, and community growth [5,6,7]. Some of the genes involved in biofilm formation are the same as those associated with host colonization. For *Vibrio* species, the presence of flagella and type IV pilus (T4P) is associated with bacterial attachment to surfaces, whether for biofilm formation or host colonization [8,9,10]. After adhesion onto a surface, bacteria begin the biofilm maturation process by producing an extracellular matrix. Different polysaccharides can be produced depending on the *Vibrio* species. The *vps* gene cluster (for *Vibrio* polysaccharide synthesis) was initially discovered in *Vibrio cholerae* but has since been identified in *Vibrio fischeri* and *Vibrio tapetis* [6,11,12]. VPS polysaccharides can constitute up to 50% of the total matrix of biofilms formed by *V. cholerae* and are mainly composed of glucose and galactose [13,14]. *Vibrio parahaemolyticus* possesses a *cps* (for capsular polysaccharides) gene locus and produces capsular polysaccharides (CPS) [15,16] involved in biofilm formation and containing various sugars such as glucose, galactose, or N-acetylglucosamine [15,17]. Polysaccharides synthesized by the products of the *syp* cluster (for symbiotic polysaccharides) in *V. fischeri* are necessary for the initiation of symbiotic relationships between *V. fischeri* and the squid *Euprymna scolopes* [18,19,20]. In a context of pathogenicity, the biofilm formation appears important for infections caused by the waterborne pathogen *V. cholerae*. There is evidence that *V. cholerae* can form aggregates similar to biofilms during infection, which may be crucial for pathogenesis and disease spread [21,22]. Indeed, biofilm growth promotes a hyperinfectious phenotype in *V. cholerae* through the up-regulation of several virulence genes [23,24]. Recently, a study demonstrated the production of biofilm around human immune cells as an aggressive cell-killing strategy [25]. 

In contrast to human pathogens, the role of the biofilm lifestyle in the virulence of *Vibrio* pathogens of marine organisms is still unclear. *Vibrio harveyi* strains are marine pathogens that affect a large range of marine vertebrates and invertebrates, inducing gastroenteritis, inflammation of the circulatory system, and skin lesions [26,27]. The *V. harveyi* ORM4 strain was the causative agent of a severe outbreak of the European abalone *Haliotis tuberculata* which occurred in natural populations and farmed stocks in the late 1990s [28]. This bacterium colonizes abalone gills, despite the presence of mucus, and was observed in aggregates on these organs, suggesting the formation of biofilm [29]. After a few hours of contact, the bacteria are found in the hemolymph of their host, where they are able to proliferate [29]. The immune depression generally associated with the abalone summer spawning period facilitates *V. harveyi* ORM4 multiplication into the hemolymph [30,31]. The mechanisms behind the bacterium’s pathogenicity against the European abalone are not well understood; a recent work demonstrated the involvement of quorum sensing (QS) and type III secretion system (T3SS) in the virulence of *V. harveyi* ORM4 but also showed that the presence of abalone serum, i.e., cell-free hemolymph, induces the production of biofilm by the bacteria [32]. These observations suggest that biofilm formation could be involved in the pathogenicity of *V. harveyi* ORM4 against European abalone. Unfortunately, no study described the molecular mechanisms involved in biofilm formation by *V. harveyi*.

Bacterial biofilm formation is generally quantified in polystyrene microplates, but this technique does not allow observation of the biofilm architecture [33]. Several other devices have been set up to grow and observe biofilms. Among these, the development of flow cell culture techniques coupled with the use of confocal laser scanning microscopy (CLSM) enables a more complete characterization, such as visualization of the three-dimensional structure, labeling of the various biofilm constituents (bacteria, matrix components), and quantification of various parameters (maximum and average thicknesses, biovolume), and allows monitoring of the biofilm formation over time [34]. The objective of the present study was to provide a comprehensive view of biofilm formation by the virulent *V. harveyi* ORM4 strain using microscopic observations coupled to a transcriptomic analysis allowing the identification of differentially expressed genes in biofilm compared to planktonic bacteria.

## 2. Materials and Methods

### 2.1. Strains and Culture Medium

*V. harveyi* ORM4 [28] was used for RNA-seq experiments and *V. harveyi* ORM4-GFP, carrying the pFD086 plasmid (Trim^R^) [32], was only used for biofilm microscopic observations. *V. harveyi* ORM4 was grown in LBS (LB containing 20 g/L NaCl) and trimethoprim (10 µg/mL) was added to maintain pFD086 inside *V. harveyi* ORM4-GFP cells.

### 2.2. Planktonic Growth

One colony of *V. harveyi* ORM4 was used to inoculate 5 mL of LBS which was incubated overnight at 20 °C with shaking. Next, 100 µL of this pre-culture was used to inoculate 5 mL LBS in triplicate and the tubes were incubated for 8 h at 20 °C with shaking. Then, 50 µL of each triplicate was used to inoculate in triplicate 20 mL LBS contained in a 100 mL Erlenmeyer flask, which was then incubated at 20 °C with shaking for 15 h (bacteria were in stationary phase). Finally, 1 mL aliquots of planktonic cells were harvested by centrifugation (6000× *g*, 6 min, 4 °C), and pellets from each culture were used for RNA extraction.

### 2.3. Biofilm Culture

*V. harveyi* ORM4 and *V. harveyi* ORM4-GFP biofilms were grown at 20 °C under hydrodynamic conditions in a three-channel flow cell (1 mm × 40 mm × 44 mm, Biocentrum, DTU, Lyngby, Denmark) [34], on a glass coverslip (24 × 50 st1 [KnittelGlasser, Braunschweig, Germany]). The system was assembled as described previously [35] and sterilized for 2 h with bleach before being washed with a flow of autoclaved distilled water overnight (running volume: 800 mL). The next day, the system was filled with LBS. A quantity of 300 µL of an overnight culture of *V. harveyi* ORM4 or *V. harveyi* ORM4-GFP diluted to an OD_600nm_ of 0.1 in artificial seawater (30 g/L sea salts [Sigma-Aldrich, Saint-Louis, MO, USA]) was injected in each channel with a 1 mL syringe and a needle (25G, 0.5 × 16 mm [Terumo, Tokyo, Japan]). An attachment step of the bacteria onto the glass surface was performed for 2 h without medium flow. After this step, LBS was applied at a flow rate of 2.5 mL/h for 24 h during which the system was incubated at 20 °C. The resulting biofilms were observed by CLSM (strain *V. harveyi* ORM4-GFP) or used to extract RNAs (strain *V. harveyi* ORM4) as described below. 

### 2.4. Confocal Laser Scanning Microscopy (CLSM)

Biofilms formed by *V. harveyi* ORM4-GFP were observed by monitoring the GFP fluorescence. Different dyes were used to observe the matrix components after biofilm growth. Polysaccharides and proteins were, respectively, stained using 100 µM Calcofluor White (Sigma Aldrich, Saint-Louis, MO, USA) [36] and 1X FilmTracer SYPRO Ruby (Invitrogen, Thermo Fisher Scientific, Waltham, MA, USA). Extracellular DNA (eDNA) was stained with 1 µM of 7-hydroxy-9H-(1,3-dichloro-9,9-dimethylacridin-2-one) (DDAO) (Invitrogen, CarIsbad, CA, USA) [37]. All dyes were prepared in artificial seawater (ASW: 30 g/L sea salts [Sigma Aldrich, Saint-Louis, MO, USA]) and staining was done by injecting in each channel 300 µL of appropriate dye(s) after biofilm growth. Biofilms were stained for 15 min in the dark in the absence of flow, before washing with an LBS flow for 10 min (2.5 mL/h). Stained biofilms were observed with a confocal laser scanning microscope (LSM 710, Zeiss, Oberkochen, Germany). GFP was excited at 488 nm and fluorescence emission was detected between 500 and 550 nm. FilmTracer SYPRO Ruby and DDAO were excited at 488 and 633 nm, and detected between 604–714 nm and 637–716 nm, respectively. These two dyes have close emission spectra that do not allow differentiating them; they could thus not be used on the same biofilms. Calcofluor White was excited at 400 nm and fluorescence emission was detected between 498 and 501 nm. Images were acquired at intervals of 1 µm throughout the whole depth of the biofilm. At least three image stacks from each eight of independent experiments (24 stacks in total) were analyzed using the COMSTAT v1 2000 software [38].

### 2.5. RNA Extraction

Total RNA was isolated for each condition (planktonic or biofilm) from three independent experiments, using the MasterPure Complete RNA purification kit (Lucigen, Biosearch Technologies, LGC, Kidlington, UK). For *V. harveyi* ORM4 planktonic cells, RNA extraction was performed from cell pellets obtained as described in Section 2.2, following the supplier’s protocol. For *V. harveyi* ORM4 biofilm cells, we followed the procedure described by Rodrigues et al., 2018 [6] to ensure that RNA was only extracted from biofilm-forming cells. Briefly, directly after microscopic observations of *V. harveyi* ORM4 biofilms, the flow was progressively increased from 2.5 mL/h to 25 mL/h for 5 min. The tubing at the end of the flow cell was subsequently cut and the content was aseptically recovered by manual flushing with 1 mL cold LBS in a syringe and placed on ice until RNA extraction. The glass slide of each flow cell was then observed to ensure the absence of residual cells. The cellular suspension was centrifuged (6000× *g*, 6 min, 4 °C) and the pellet was used for RNA extraction. The amount and quality of total RNA were assessed using a NanoPhotometer N60 (Implen, Munich, Germany) spectrophotometer. 

### 2.6. RNA-Sequencing and Data Analysis

Ribosomal RNA depletion, cDNA library preparation, and Illumina sequencing were performed by GATC laboratory (Eurofins, GATC Biotech, Konstanz, Germany).

Raw data analysis was performed using the Galaxy France platform (Galaxy France, https://usegalaxy.fr, accessed on September 2022). Raw data quality was analyzed with the FastQC tool (version 0.73) [39] and sequence cleaning was performed with the Trimmomatic tool (version 0.38.1) [40]. The sequences were then mapped onto the *V. harveyi* ORM4 genome (GenBank assembly accession number GCA_963920535) using the Boowtie2 software (Version 2.4.5) [41]. The abundance of each transcript was calculated using the union model with the HTseq-count tool (Version 0.9.1) [42]. Subsequently, statistical analysis of the differentially expressed genes was performed with the R packages SARTools and DESeq2 [43]. These packages allow data normalization and differential expression tests for each feature between conditions and raw *p*-value adjustment. Genes were considered significantly differentially expressed if the *p*-value adjusted (*p*adj) by False Discovery Rate (FDR) correction [44] was less than 0.05. The raw data obtained in this study have been deposited in the SRA database and are accessible under PRJNA1010657 accession number. The RNA-seq data were plotted on a volcano plot with the negative log of the adjusted *p*-value on the y axis and the log2 of the fold change between the biofilm and planktonic condition on the x axis using the ggplot2 v3.4.4 package [45]. R version 4.2.0 was used for all analysis.

### 2.7. mRNA Quantification by Reverse Transcription Followed by Quantitative PCR (RT-qPCR)

The RNA-seq results were validated by RT-qPCR assays. Total RNA was converted to cDNA using the High Capacity cDNA Reverse Transcriptase Kit (Applied Biosystems, Foster City, CA, USA). Eight genes were chosen for which primers (Table 1) were designed using the Primer Express v3 software (Applied Biosystems, Foster City, CA, USA). qPCR reactions were then performed with a 7300 RT PCR System (Applied Biosystems), using Master Mix PCR SYBR Green (Applied Biosystems). Gene level expression was obtained by the comparative CT (2^−∆∆CT^) method [46], using 16S ribosomal RNA (rRNA) as endogenous control.

## 3. Results and Discussion

### 3.1. Biofilm Formation by V. harveyi

#### 3.1.1. *V. harveyi* ORM4-GFP Biofilm Presents a Non-Uniform Structure

**Figure 1:**
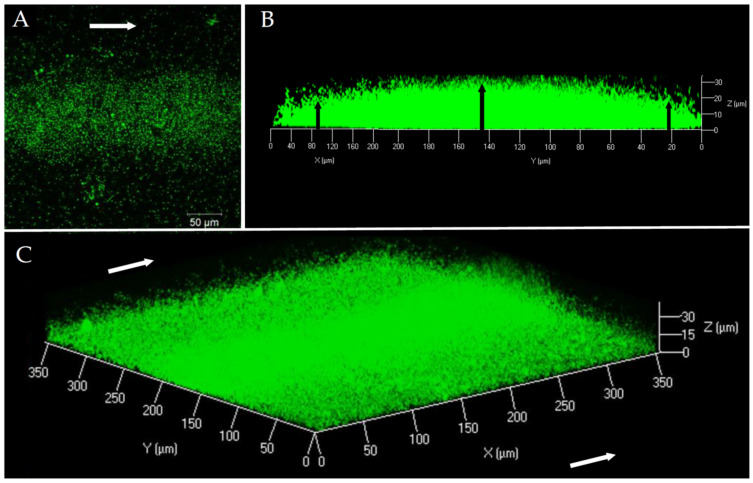
*V. harveyi* ORM4-GFP biofilm observation at 40× magnification by CLSM after 24 h of culture at 20 °C under a constant flow of LBS (2.5 mL/h). Top view (**A**), side view (**B**), and three-dimensional view (**C**). White arrows represent the LBS flow direction and black arrows indicate the difference in biofilm thickness between the center and the ends.

In order to characterize the structure and composition of the biofilm of a pathogenic *V. harveyi* strain, the ORM4-GFP strain was grown in a flow cell system. The first microscopic observations showed that *V. harveyi* ORM4-GFP was able to produce a biofilm on a glass slide in the presence of a constant LBS flow (2.5 mL/h) during 24 h at 20 °C (Figure 1). Our observations highlighted a curved structure (convex shape) of the biofilm: the highest biofilm thickness was observed in the center of the glass slide, in the same orientation as the LBS flow (Figure 1B). On both sides, the biofilm thickness was decreased (Figure 1B) and bacteria were found in monolayer until their total absence on the edges of the glass slide [47].

This biofilm structure was found on the whole length of the flow cell channel. Biofilms of *V. harveyi* ORM4-GFP reached a biovolume of 5.4 ± 0.7 µm^3^/µm^2^ and a maximal thickness of 27.5 ± 1.7 µm. However, due to this curved structure, the average thickness was only 9.5 ± 1 µm. Such a structure is unusual since we could not find any other example in the literature. Actually, CLSM observations of biofilms formed under the same conditions by other *Vibrio* species revealed biofilms that uniformly covered the surfaces [48,49].

#### 3.1.2. Matrix Component Distribution

**Figure 2:**
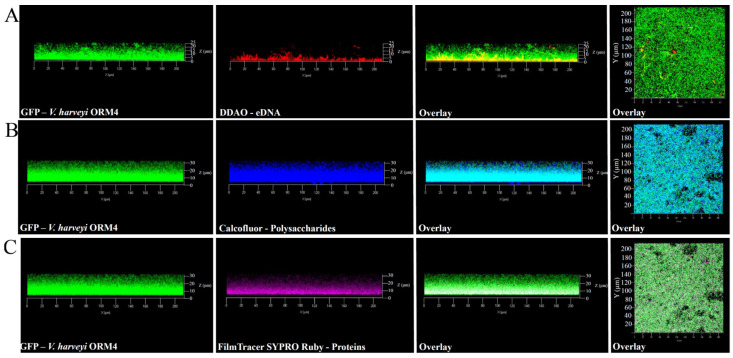
Observations with the CLSM at 40× magnification of matrix components of 24 h *V. harveyi* ORM4-GFP biofilms. The matrix components were stained using the following dyes: eDNA with DDAO (**A**), polysaccharides with Calcofluor (**B**), and proteins with FilmTracer SYPRO Ruby (**C**). The right panels show a top view of the biofilm.

In addition to the observation of *V. harveyi* ORM4-GFP biofilm-forming cells, the exploration of the biofilm matrix produced by *V. harveyi* ORM4-GFP cells was performed with specific dyes allowing the visualization of matrix components. Biofilms were stained with Calcofluor White (which binds to *β*1–3 and *β*1–4 polysaccharides), DDAO (which binds to eDNA), and FilmTracer SYPRO Ruby (which binds to proteins). CLSM observations enabled the visualization of the distribution of these different components surrounding the bacterial cells. *β*-polysaccharides and proteins were observed across the entire biofilm thickness (Figure 2B,C), while eDNA were mostly localized in the lower half of the biofilm (Figure 2A). The presence of these different components was previously reported in *V. cholerae*, *V. parahaemolyticus*, and *V. tapetis* biofilms [11,48,49,50,51].

Although *V. harveyi* ORM4-GFP forms biofilms with a curved structure, this does not appear to be induced by matrix structuring since the matrix components were observed across the entire width of the biofilm (Figure 2). Several hypotheses can be put forward to explain this particular biofilm structure, such as the nature of the substrate, the availability of nutrients, and the pH [52,53,54]. However, *V. harveyi* ORM4-GFP cells grown under static conditions in glass-bottom microplates and in the same medium yielded uniformly thick biofilms which evenly covered the glass surface at the bottom of the wells [47]. Altogether, this suggests that the flow may induce the curved structure of the biofilm and that this particular biofilm structure did not result from a non-homogeneous accumulation of the matrix.

### 3.2. Transcriptomic Comparison between Planktonic and Biofilm Cells

**Figure 3:**
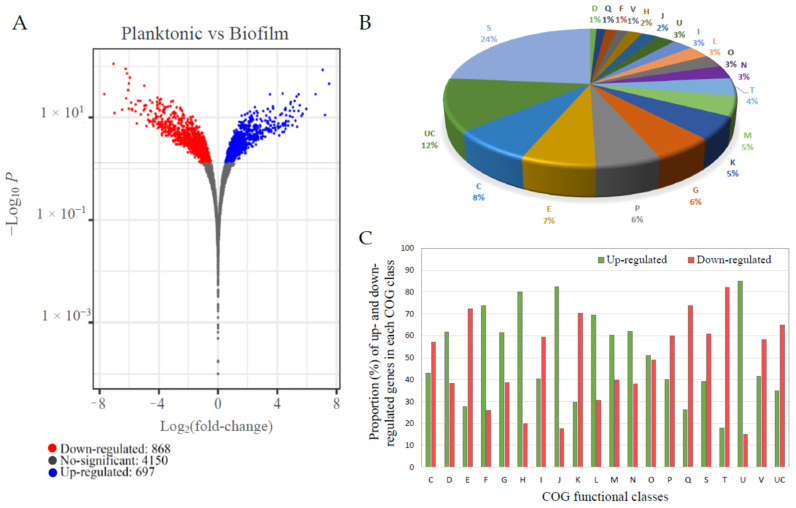
Summary of the *V. harveyi* ORM4 RNA-seq analysis results. Volcano plot representation of differentially expressed genes (DEGs) in *V. harveyi* ORM4 biofilm cells versus planktonic cells. The blue and red dots represent significantly up- and down-regulated genes (adjusted *p*-value < 0.05), respectively, and the grey dots represent the non-differentially expressed genes (adjusted *p*-value > 0.05) (**A**). Distribution of DEGs in *V. harveyi* ORM4 biofilm cells in functional classes (COG) (**B**). Percentage of up- and down-regulated genes for each COG class in biofilm cells versus planktonic cells (**C**). COG classes: C: Energy production and conversion. D: Cell cycle control, cell division, and chromosome partitioning. E: Amino acid transport and metabolism. F: Nucleotide transport and metabolism. G: Carbohydrate transport and metabolism. H: Coenzyme transport and metabolism. I: Lipid transport and metabolism. J: Translation, ribosomal structure, and biogenesis. K: Transcription. L: Replication, recombination, and repair. M: Cell wall/membrane/envelope biogenesis. N: Cell motility. O: Posttranslational modification, protein turnover, and chaperones. P: Inorganic ion transport and metabolism. Q: Secondary metabolite biosynthesis, transport, and catabolism. S: Function unknown. T: Signal transduction mechanisms. U: Intracellular trafficking, secretion, and vesicular transport. V: Defense mechanisms. UC: Unclassified.

In order to obtain an insight into the molecular mechanisms behind biofilm formation by *V. harveyi* ORM4, an RNA-seq transcriptome analysis was performed using *V. harveyi* ORM4 planktonic cells as the control group and *V. harveyi* ORM4 biofilm cells as the experimental group. Analysis of 12 million sequences obtained for each condition revealed that 1565 genes (29% of the total *V. harveyi* ORM4 genes) were significantly differentially expressed (adjusted *p*-value < 0.05), out of which 868 and 697 were, respectively, down- and up-regulated in biofilm cells (Figure 3A). 

**Figure 4:**
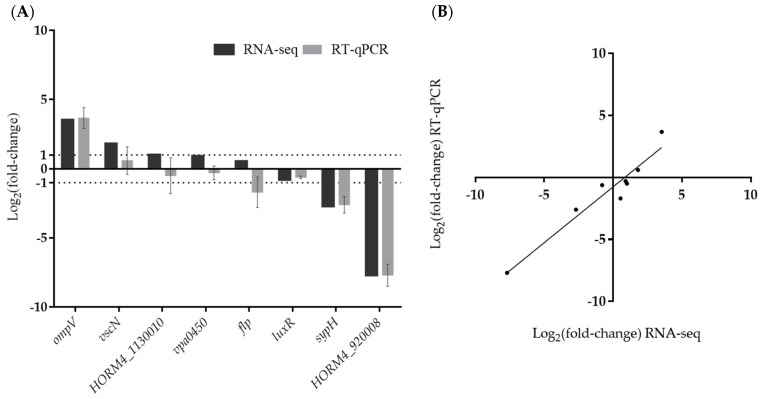
Validation of RNA-seq data by RT-qPCR. Comparison of log_2_(fold-change) (log_2_FC) gene expression values obtained in RT-qPCR and RNA-seq (**A**). Correlation of log_2_FC values determined by RNA-seq and RT-qPCR in biofilm-forming *V. harveyi* ORM4 versus planktonic cells (**B**).

In order to validate the results obtained by RNA-seq analysis, we selected eight genes involved in biofilm formation (*flp*, *luxR*, and *sypH*) [18,55,56], virulence (*ompV*, *vscN*, and *vpa0450*) [57,58,59], or encoding unknown proteins (HORM4_920008, HORM4_1130010) and their relative expression levels (Figure 4) were evaluated using RT-qPCR. These genes showed a wide range of fold-change values in RNA-seq, and were up-regulated (*vscN*, *vpa0450* and *ompV*), down-regulated (*luxR*, *sypH* and HORM4_920008), or not differentially expressed (*flp* and HORM4_1130010). These eight genes were found to have high Pearson correlation coefficients (0.93) between their fold-change levels assessed by RT-qPCR and by RNA-seq, thereby validating the RNA-seq findings (Figure 4B). 

### 3.3. Differential Expression Analysis of Biofilm- and Virulence-Related Genes

To obtain further insights into biofilm formation by a pathogenic strain of *V. harveyi*, and into the possible links between biofilm and virulence, we then focused our analysis of the RNA-seq data on genes encoding proteins which could be associated with these two phenotypes.

#### 3.3.1. DEGs Involved in Motility and Attachment

**Figure 5:**
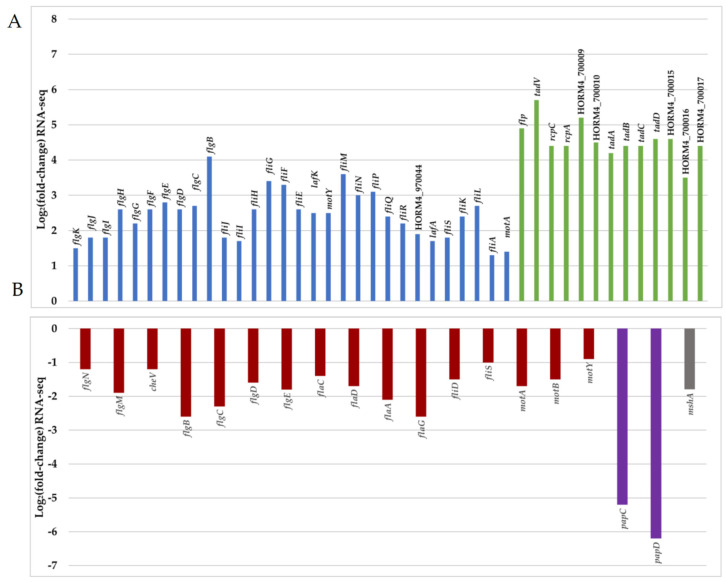
Representation of Log_2_FC values of 64 DEGs (adjusted *p*-value < 0.05) related to motility and attachment in *V. harveyi* ORM4 biofilm cells versus planktonic cells. Positive (**A**) and negative (**B**) values of Log_2_FC are represented in two separated graphics. Red bar represents DEGs encoding the polar flagellum, blue bar represents DEGs encoding lateral flagella, green bar represents DEGs encoding a putative Tad pilus (gene cluster HORM4_700005 to HORM4_700017), and purple and grey bars represent, respectively, DEGs encoding the P pilus (HORM4_920006 and HORM4_920007) and the MSHA pilin protein (HORM4_370020).

A necessary step prior to successful biofilm formation or infection is the ability to swim and adhere to surfaces. Bacteria switch from a planktonic swimming state, associated with the presence of a polar flagellum, to attachment via filamentous appendages such as lateral flagella or pili. A total of 3% of the DEGs were associated with motility (COG class N), among which 62% were up-regulated in biofilm-forming cells (Figure 3B,C). In biofilm cells, genes encoding protein forming key parts of the polar flagellum (hook, basal body, and motor) were significantly down-regulated, while lateral flagellum genes were up-regulated (Figure 5 and Table S1). The lack of transcriptional activity linked with swimming motility involving the polar flagellum in mature biofilm cells has been previously described for *Pseudomonas aeruginosa* [60,61], which is in agreement with our observations. In parallel, bacteria attach to surfaces by the production of proteinaceous appendages such as pili. In *Vibrio* species, several types of pili are involved in early attachment to abiotic surfaces [12]. In the genome of *V. harveyi* ORM4, we found two sets of genes encoding type IV pili. The first one is a gene locus (HORM4_370011 to HORM4_370026) homologous to the gene cluster for the mannose-sensitive haemagglutinin pilus (MSHA pilus) of *V. cholerae* O1 El Tor [62], and the second one includes genes similar to the genes encoding the PapC porin and the PapD chaperone of the P pilus of *V. parahaemolyticus* RIMD 2210633 (HORM4_920006 and HORM4_920007, respectively).

In *V. cholerae*, the MSHA pilus plays important roles in the persistence of the bacteria by mediating their attachment to nutritive substrates, in biofilm formation, and in chitin utilization [63,64]. *V. parahaemolyticus* also produces an MSHA pilus, which is used to anchor the bacteria to chitinous surfaces or produce biofilms, whereas the P pilus is associated with host adhesion [8,65]. In *V. harveyi* ORM4 biofilm cells, the HORM4_370020 gene encoding the pilin protein MshA is 3.8-fold down-regulated and the genes encoding PapC and PapD were 46 to 100-fold down-regulated (log_2_FC between −6.2 and −5.2) (Figure 5 and Table S1). Although the encoding genes are down-regulated in mature biofilms, the MSHA and P pili could participate in the *V. harveyi* ORM4 anchoring to the glass slide during an earlier stage of biofilm formation, since stained proteins are localized at the biofilm basis (Figure 2). This would be consistent with previous reports showing that *Vibrio* bacteria defective in the production of these pili failed to adhere or to progress from the monolayer formation to three-dimensional biofilms [17,66,67]. 

An expert annotation of the *V. harveyi* ORM4 genome was done using the MaGe tool of the Microscope platform [68], leading to the identification of three distinct loci putatively encoding a tight adherence (Tad) pilus (Table S2). One of these loci (HORM4_700005 to HORM4_700017) is fully induced in *V. harveyi* ORM4 biofilm cells, with up to 53-fold (log_2_FC between 3.5 and 5.7) induction levels (Figure 5 and Table S1). Genes homologous to Tad pilus biogenesis genes are widespread in *Vibrionaceae* [69]. In *V. vulnificus*, the Tad pilus has been shown to be involved both in adhesion and in biofilm maturation since cells lacking the Tad pilin required more time to attach to a surface and to form a biofilm of a substantial biomass [55,70]. Concerning *V. harveyi* ORM4, our transcriptomic results suggested the involvement of the Tad pilus in the process of biofilm maturation, possibly in its structuration as is the case for the type IV pili in *P. aeruginosa* biofilm or fimbriae in *E. coli* and *Salmonella enterica* serovar Typhimurium biofilms [71,72].

#### 3.3.2. DEGs Involved in Polysaccharide Production

Staining of the biofilm matrix revealed the presence of *β*1–3 and *β*1–4 polysaccharides on the whole thickness of *V. harveyi* ORM4 biofilm (Figure 2). Several studies have reported the involvement of *vps* and *syp* gene clusters in *Vibrio* biofilm formation [11,12,20]. Within the *V. harveyi* ORM4 genome, we identified a gene cluster (HORM4_940043 to HORM4_940054) which encodes proteins sharing over 67% amino acid identities with the proteins produced by the *V. parahaemolyticus cps* cluster (vpa1403 to vpa1413) [15] (Table S2). However, there was no significant difference in their expression between biofilm and planktonic conditions (adjusted *p*-value ≥ 0.05) (Table S3). Nevertheless, we furthermore found in the *V. harveyi* ORM4 genome a gene cluster homologous to the *syp* cluster, initially identified in *V. fischeri* but conserved in many pathogenic *Vibrio* species such as *V. parahaemolyticus*, *V. tapetis*, and *V. vulnificus* (Table 2) [6,12]. In the *V. harveyi* ORM4 genome, all the *syp* genes were found (HORM4v2_640011 to HORM4_640026), except the genes encoding SypE, SypF, and SypM. SypE and SypF are both regulatory proteins [73,74,75], while the role of SypM remains to be defined. Among the 15 identified *syp* genes, 5 were down-regulated in *V. harveyi* ORM4 biofilm cells (Table 2). 

Our transcriptomic analysis revealed that, despite the presence of polysaccharide synthesis genes, these genes are either not more expressed or down-regulated in *V. harveyi* ORM4 after 24 h of biofilm formation compared to the planktonic condition. These results suggest that if an over-production of polysaccharides occurs in the process of biofilm formation by *V. harveyi* ORM4, it likely occurs earlier. This would be consistent with previous reports showing that the synthesis of polysaccharides starts during the adhesion step [76,77].

#### 3.3.3. Genes Involved in Quorum Sensing

In bacteria, the control of gene expression in response to cell population density is known as quorum sensing (QS) [78,79]. In the *V. harveyi* ORM4 genome, we were able to identify genes encoding the three QS systems previously described in *Vibrio campbellii*: *luxM*/*luxN*, *luxS*/*luxPQ*, and *cqsA/cqsS* [56]. Among these genes, *cqsS* was weakly down-regulated (log_2_FC: −0.7) in *V. harveyi* ORM4 biofilm cells, whereas the other genes were not differentially expressed (Table 3). In *V. campbellii*, the different QS systems converge to the transcriptional regulator proteins LuxO and LuxR [56]. *V. harveyi* ORM4 possesses both a *luxO* [32] and a *luxR* gene. Our data showed that both *luxO* and *luxR* were weakly down-regulated (log_2_FC: −0.8 for both genes) in *V. harveyi* ORM4 biofilm cells (Table 3). In *V. tapetis* or *V. parahaemolyticus* biofilms, transcriptomic studies described the induction of QS-related genes [6,7]. In contrast to these species, the expression of *V. harveyi* ORM4 QS-related genes was found to be weakly down-regulated or unchanged in comparison with planktonic cells (Table 3), suggesting a low QS activity in *V. harveyi* ORM4 biofilm cells. It remains possible that an up-regulation of these genes occurs earlier in the process of biofilm formation. 

#### 3.3.4. Type III Secretion System (T3SS)-Associated Genes

In a previous study [32], we identified a 33-kb genetic region homologous to the region encoding the T3SS1 in *V. parahaemolyticus* [79,80]. The deletion of *exsA*, encoding the major regulator of the T3SS1 genes, abolished the bacterial virulence against European abalones [32]. Our RNA-seq analysis revealed an up-regulation (two- to four-fold) of 18 of the 49 T3SS genes in biofilm cells (Table 4). Proteins encoded by these genes are part of the structure of the T3SS or are involved in its functioning. This includes genes encoding the VscC, VscD, VscI, VscJ, VscT, VscR, and VscQ proteins, which compose the basal body of the T3SS and are located on the bacterial inner and outer membranes [58,81]. In addition, *vscN* encoding an ATPase and the *vscF* gene enabling synthesis of the needle found in the extracellular space were also over-expressed (Table 4) [58,82]. On the other hand, genes encoding the ExsA, ExsC, ExsD, and ExsE proteins, which compose the cascade of the T3SS regulation, were not differentially expressed. Genes encoding the VopB and VopD proteins, required for translocation of T3SS effectors across the host cell membrane, showed no difference in expression between the two culture conditions [83]. These results suggest that the T3SS is assembled and that the biofilm lifestyle induces the T3SS in *V. harveyi* ORM4. To our knowledge, this is the first time that the potential involvement of T3SS in bacterial biofilm formation has been suggested. This secretion system is mainly associated with bacterial virulence [10,32,58]. It is therefore surprising to observe an induction of T3SS genes in a monospecies *V. harveyi* biofilm. Induced genes encode structural proteins that make up the basal body and the needle found in the extracellular environment [58,82]. If the T3SS needle is produced, it is possible that the apparatus is stained by the FilmTracer SYPRO Ruby dye we used to stain the *V. harveyi* ORM4 biofilm matrix (Figure 2).

Among the secretion systems, the type VI secretion system (T6SS) was the only one described as being involved in both biofilm production and bacterial virulence. Many bacterial pathogens, including *P. aeruginosa* and *V. cholerae*, are known to use their T6SS during infection [84]. Some studies have shown an induction of the expression of T6SS genes in biofilms of *P. aeruginosa* or *V. tapetis*, including genes encoding T6SS effectors [6,85]. Gallique et al. hypothesized that secretion of T6SS proteins in *Pseudomonas fluorescens* biofilm could participate in cell communication [86]. This hypothesis cannot be made in the case of T3SS since, to our knowledge, only eukaryotic cells are targeted by effectors [84,87,88,89]. Even if its role remains unknown in biofilm formation, it is important to note that T3SS genes are induced by this lifestyle in *V. harveyi* ORM4 cells. The infection of European abalone by *V. harveyi* ORM4 starts by the colonization of the abalone gills on which the bacteria were observed in aggregates [29]. Here, the induction of T3SS genes in *V. harveyi* ORM4 biofilm may suggest that this lifestyle could be the first step of the infection. 

## 4. Conclusions

In summary, this is the first report describing a *V. harveyi* biofilm and the molecular changes that occurred during the biofilm production. In mature *V. harveyi* biofilms, the gene expression pattern was largely modified compared to planktonic stationary phase bacteria since 29% of the genes were differentially expressed, and these DEGs were assigned to various functional classes. Among the functions related to biofilm formation, the impairment of swimming motility was indicated by the down-regulation of polar flagellum-related genes. Our transcriptomic analysis revealed a 53-fold induction of the gene Tad pilus in *V. harveyi* ORM4 cell biofilm, a type IV pilus involved in both biofilm formation and host colonization in *V. vulnificus* [55,70]. Finally, we also found that genes for T3SS are up-regulated while earlier work demonstrated its requirement for *V. harveyi* virulence [32], suggesting that the biofilm formation might be involved in the infection of European abalone by *V. harveyi*. Future work will be undertaken to study the role of T3SS in biofilm formation. 

## Figures and Tables

**Table 1:** Primers used in RT-qPCR. Primers are oriented 5′ to 3′.

Primer Name	5′-3′ Sequence	Amplified Gene (ID)
ARNr16S-F	TGCGCTTTACGCCCAGTAAT	rRNA 16S
ARNr16S-R	GGTAATACGGAGGGTGCGAG	
flp-F	CGAGGTGTAACCGCTGTTGAA	*flp* (HORM4_610121)
flp-R	TGATGACATTGCAACAGCGA	
Vscn-F	CCTCGTCGTGTTGGTGGTTC	*vscN* (HORM4_240127)
Vscn-R	AGCTCAGCGTGAGATTGGCT	
VPA0450-F	TCGCTGAGGTCACATCATCAA	*vpa0450* (HORM4_520123)
VPA0450-R	AGCCTGATACTGATCCGGCA	
luxR-F	TGTTTTGCACCAGCAGTTGG	*luxR* (HORM4_420032)
luxR-R	GGCCGCTATTCGTAACGACA	
ompV-F	CATCGTTGTCACCTAGGAAACG	*ompV* (HORM4_1070102)
ompV-R	TCAACGCTGATTTAGGCGGT	
sypH-F	CGTCAAGGATGAGCCTTACGA	*sypH* (HORM4_640016)
sypH-R	GCCTCACGTCCCGTTTCTAC	
920008-F	GACCTTCGTCAGACCCAACG	HORM4_920008
920008-R	TATCGGCTGGTGCAGTTGC	
1130010-F	TCAAATAGAAGAGTTGGCTGCG	HORM4_1130010
1130010-R	GGACCAAAAATCCCTTTCACG	

**Table 2:** *syp* gene expression in *V. harveyi* ORM4 biofilm cells (adjusted *p*-value < 0.05).

Gene ID	Gene Name	COG	FC	Log_2_FC	Product
HORM4_640012	*sypB*	M	0.1	−3	Outer membrane protein
HORM4_640013	*sypC*	M	0.2	−2.7	Polysaccharide export periplasmic protein
HORM4_640014	*sypD*	M	0.2	−2	Chromosome partitioning ATPase
HORM4_640016	*sypG*	T	0.2	−2.7	Sigma-54 dependent transcriptional regulator
HORM4_640017	*sypH*	M	0.3	−2	Glycosyl transferases group 1

**Table 3:** Differential expression of genes involved in quorum sensing when *V. harveyi* ORM4 produced biofilm in comparison with planktonic cells.

Gene ID	Gene Name	COG	FC	Log_2_FC	Product
HORM4_830068	*cqsA*	E	Not DEG		CAI-1 autoinducer synthase
HORM4_830069	*cqsS*	T	0.6	−0.7	CAI-1 autoinducer sensor kinase/phosphatase CqsS
HORM4_530027	*luxM*	T	Not DEG		Acyl-homoserine-lactone synthase LuxM
HORM4_530026	*luxN*	T	Not DEG		Autoinducer 1 sensor kinase/phosphatase LuxN
HORM4_420009	*luxS*	T	Not DEG		S-ribosylhomocysteinase
HORM4_930034	*luxP*	G	Not DEG		Autoinducer 2-binding periplasmic protein LuxP
HORM4_930033	*luxQ*	T	Not DEG		Autoinducer 2 sensor kinase/phosphatase LuxQ
HORM4_520007	*luxU*	T	Not DEG		Phosphorelay protein LuxU
HORM4_520006	*luxO*	T	0.6	−0.8	Transcriptional regulator LuxO
HORM4_420032	*luxR*	K	0.6	−0.8	Transcriptional regulator LuxR

**Table 4:** Differentially expressed genes belonging to the T3SS in *V. harveyi* ORM4 biofilm cells.

Gene ID	Gene Name	COG	FC	Log_2_FC	Product
HORM4_240099	*vscB*	O	3.3	1.7	T3SS chaperone, YscB family
HORM4_240100	*vscC*	U	2.7	1.4	T3SS secretin (VscC)
HORM4_240101	*vscD*	S	2.2	1.1	T3SS inner membrane ring protein (VscD)
HORM4_240103	*vscF*	U	2.8	1.5	Needle major subunit (VscF)
HORM4_240104	*vscG*	S	3.1	1.6	T3SS chaperone (VscG)
HORM4_240105	*vscH*	S	3.8	1.9	T3SS effector, YopR family
HORM4_240106	*vscI*	S	3.1	1.6	T3SS inner rod protein (VscI)
HORM4_240107	*vscJ*	U	2.1	1.1	T3SS bridge between inner and outer membrane lipoprotein (VscJ)
HORM4_240109	*vscL*	N	2.7	1.4	Yop proteins translocation protein L (VscL)
HORM4_240121	*vscT*	U	1.9	0.9	T3SS inner membrane protein (VscT)
HORM4_240123	*vscR*	U	2	1	T3SS inner membrane protein (VscR)
HORM4_240124	*vscQ*	N	2.6	1.4	T3SS inner membrane protein (VscQ)
HORM4_240126	*vscO*	U	2.3	1.2	Putative T3SS chaperone (VscO)
HORM4_240127	*vscN*	N	3.5	1.8	ATPase (VscN)
HORM4_240128	*yopN*	S	2.1	1.1	T3SS secretion regulator (YopN)
HORM4_240130	*sycN*	S	2	1	T3SS chaperone (SycN)
HORM4_240131	*vscX*	U	2.6	1.4	Putative type III secretion protein (VscX)
HORM4_240133	*vcrD*	U	2.8	1.5	Low calcium response protein (VcrD)

## Data Availability

The RNA-seq raw data obtained in this study are available at the NCBI Sequence Read Archive (SRA) under BioProject PRJNA1010657 (sample accession numbers: SAMN37193765, SAMN37193766, SAMN37193767, SAMN37193768, SAMN37193769 and SAMN37193770).

## Supplementary Materials

The following supporting information can be downloaded at: https://www.mdpi.com/article/10.3390/microorganisms12010186/s1, Table S1: Expression of genes involved in motility and attachment in *V. harveyi* ORM4 biofilm cells (adjusted *p*-value < 0.05); Table S2: *V. harveyi* ORM4 Tad proteins and amino acidic percentage of identity with Tad proteins of *V. vulnificus* CMCP6. Table S3: *V. harveyi* ORM4 CPS proteins and amino acidic percentage of identity with CPS proteins of *V. parahaemolyticus* RIMD2210633; Table S4: Expression of genes associated to the *cps* cluster in *V. harveyi* ORM4 biofilm-forming cells.

## Abbreviations

Capsular polysaccharides (cps), cluster of orthologous genes (COG), confocal laser scanning microscopy (CLSM), 7-hydroxy-9H-(1,3-dichloro-9,9-dimethylacridin-2-one) (DDAO), differentially expressed genes (DEGs), extracellular DNA (eDNA), green fluorescent protein (GFP), lysogeny broth (LB), mannose-sensitive haemagglutinin pilus (MSHA), *p*-value adjusted (padj), quorum sensing (QS), ribosomal RNA (rRNA), symbiotic polysaccharides (syp), tight adherence (Tad), trimethoprim resistance (TrimR), type IV pilus (T4P), type III secretion system (T3SS), type VI secretion system (T6SS), viable but non-culturable state (VBNC), Vibrio polysaccharide synthesis (vps).
