# Supplementary material for: Genetic Insights into Biofilm Formation by a Pathogenic Strain of Vibrio harveyi
Source: Microorganisms. 2024 Jan 17;12(1):186. doi: 10.3390/microorganisms12010186 (PMC10820411; doi:10.3390/microorganisms12010186)
Supplement: Supplementary file 1 [file microorganisms-12-00186-s001.zip › microorganisms-2764938-supplementary.pdf]

Table S1. Expression of genes involved in motility and attachment in *V. harveyi* ORM4 biofilm cells (adjusted *p*-value < 0.05).

Vibrio harveyi ORM4 gene ID	Gene Name	FC	Log_2FC	Product
**HORM4_180020**	*flgN*	0.424	-1,2	Flagellar biosynthesis protein FlgN
**HORM4_180021**	*flgM*	0.27	-1,9	Negative regulator of flagellin synthesis FlgM
**HORM4_180023**	*cheV*	0.445	-1,2	Chemotaxis protein CheV
**HORM4_180026**	*flgB*	0.166	-2,6	Flagellar basal body rod protein FlgB
**HORM4_180027**	*flgC*	0.206	-2,3	Flagellar basal body rod protein FlgC
**HORM4_180028**	*flgD*	0.336	-1,6	Flagellar basal body rod modification protein FlgD
**HORM4_180029**	*flgE*	0.29	-1,8	Flagellar hook protein FlgE
**HORM4_180037**	*flaC*	0.373	-1,4	Flagellin protein FlaC
**HORM4_180038**	*flaD*	0.315	-1,7	Flagellin protein FlaD
**HORM4_460050**	*flaA*	0.236	-2,1	Flagellin protein FlaA
**HORM4_460051**	*flaG*	0.16	-2,6	Flagellar protein FlaG
**HORM4_460052**	*fliD*	0.342	-1,5	Flagellar hook-associated protein FliD
**HORM4_460054**	*fliS*	0.492	-1,0	Flagellar secretion chaperone FliS
**HORM4_170051**	*motA*	0.303	-1,7	Flagellar motor rotation protein MotA
**HORM4_170052**	*motB*	0.346	-1,5	Flagellar motor rotation protein MotB
**HORM4_510008**	*motY*	0.528	-0,9	Sodium-type flagellar protein MotY precursor
**HORM4_1020003**	*flgK*	2.785	1,5	Flagellar hook-associated protein FlgK
**HORM4_1020004**	*flgJ*	3.524	1,8	Peptidoglycan hydrolase FlgJ
**HORM4_1020005**	*flgI*	3.529	1,8	Flagellar P-ring protein FlgI
**HORM4_1020006**	*flgH*	6.099	2,6	Flagellar L-ring protein FlgH
**HORM4_1020007**	*flgG*	4.624	2,2	Flagellar basal body rod protein FlgG
**HORM4_1020008**	*flgF*	6.017	2,6	Flagellar basal body rod protein FlgF
**HORM4_1020009**	*flgE*	7.182	2,8	Flagellar hook protein FlgE
**HORM4_1020010**	*flgD*	6.211	2,6	Flagellar basal body rod modification protein FlgD
**HORM4_1020011**	*flgC*	6.328	2,7	Flagellar basal body rod protein FlgC
**HORM4_1020012**	*flgB*	16.766	4,1	Flagellar basal body rod protein FlgB
**HORM4_970028**	*fliJ*	3.437	1,8	Flagellar export protein FliJ
**HORM4_970029**	*fliI*	3.184	1,7	Flagellum-specific ATP synthase FliI
**HORM4_970030**	*fliH*	5.9	2,6	Flagellar assembly protein FliH
**HORM4_970031**	*fliG*	10.341	3,4	Flagellar motor switch protein FliG
**HORM4_970032**	*fliF*	9.928	3,3	Flagellar M-ring protein FliF
**HORM4_970033**	*fliE*	6.046	2,6	Flagellar hook–basal body complex protein FliE
**HORM4_970034**	*lafK*	5.743	2,5	Sigma-54 dependent transcriptional regulator LafK
**HORM4_970035**	*motY*	5.752	2,5	Component of sodium-driven polar flagellar motor MotY
**HORM4_970037**	*fliM*	11.904	3,6	Flagellar motor switch protein FliM
**HORM4_970038**	*fliN*	8.238	3,0	Flagellar motor switch protein FliN
**HORM4_970039**	*fliP*	8.843	3,1	Flagellar biosynthesis protein FliP
**HORM4_970040**	*fliQ*	5.388	2,4	Flagellar biosynthesis protein FliQ
**HORM4_970041**	*fliR*	4.438	2,2	Flagellar biosynthesis protein FliR
**HORM4_970044**		3.666	1,9	Conserved protein of unknown function
**HORM4_970045**	*lafA*	3.34	1,7	Lateral flagellin LafA
**HORM4_980002**	*fliS*	3.493	1,8	Flagellar secretion chaperone fliS
**HORM4_980004**	*fliK*	5.265	2,4	Flagellar hook-length control protein FliK
**HORM4_980005**	*fliL*	6.452	2,7	Flagellar basal body-associated protein FliL

**HORM4_980006**	*fliA*	2.412	1,3	RNA polymerase sigma factor for flagellar operon FliA
**HORM4_980007**	*motA*	2.576	1,4	Flagellar motor rotation protein MotA
**HORM4_700005**	*flp*	29.13	4,9	Flp pilus assembly protein, pilin Flp
**HORM4_700006**	*tadV*	52.965	5,7	Type IV prepilin peptidase TadV
**HORM4_700007**	*cpaB*	21.33	4,4	Flp pilus assembly protein CpaB
**HORM4_700008**	*cpaC*	20.681	4,4	Type II/IV secretion system secretin CpaC, associated with Flp pilus assembly
**HORM4_700009**		37.605	5,2	Conserved exported protein of unknown function
**HORM4_700010**		23.155	4,5	Conserved protein of unknown function
**HORM4_700011**	*tadA*	18.403	4,2	TadA type II/IV secretion system ATP hydrolase
**HORM4_700012**	*tadB*	20.76	4,4	Flp pilus assembly protein TadB
**HORM4_700013**	*tadC*	20.825	4,4	Type II/IV secretion system protein TadC, associated with Flp pilus assembly
**HORM4_700014**	*tadD*	24.047	4,6	Flp pilus assembly protein TadD
**HORM4_700015**		23.852	4,6	TadE-like protein
**HORM4_700016**		11.177	3,5	Conserved protein of unknown function
**HORM4_700017**		21.176	4,4	Conserved protein of unknown function
**HORM4_920006**	*papC*	0.026	-5,2	P pilus assembly protein, porin PapC
**HORM4_920007**	*papD*	0.014	-6,2	P pilus assembly protein, chaperone PapD
**HORM4_370020**	*mshA*	0.289	-1,793	MSHA pilin protein MshA

Table S2. *V. harveyi* ORM4 Tad proteins and amino acidic percentage of identity with Tad proteins of *V. vulnificus* CMCP6.

Vibrio harveyi **ORM4 gene ID**	**% aa identity**	***Vibrio vulnificus*** **CMCP6 gene ID**	***Vibrio vulnificus*** **CMCP6 protein**
**HORM4_700005**	41,5	vv1_3224	Flp
**HORM4_700006**	29,5	vv1_2329	TadV
**HORM4_700007**	23,64	vv1_2330	RcpC
**HORM4_700008**	31,14	vv1_2331	RcpA
**HORM4_700011**	51,8	vv1_2334	TadA
**HORM4_700012**	30,2	vv1_2335	TadB
**HORM4_700013**	24,9	vv1_2336	TadC
**HORM4_700014**	22,9	vv1_2337	TadD
**HORM4_610121**	46,2	vv2_0084	Flp
**HORM4_610122**	44,6	vv2_0085	RcpC
**HORM4_610123**	71,9	vv2_0086	RcpA
**HORM4_610124**	32,2	vv2_0087	RcpB
**HORM4_610125**	56,2	vv2_0088	TadZ
**HORM4_610126**	86	vv2_0089	TadA
**HORM4_610127**	63,3	vv2_0090	TadB
**HORM4_610128**	56,6	vv2_0091	TadC
**HORM4_610129**	53,7	vv2_0092	TadD
**HORM4_610130**	42,8	vv2_0093	TadE
**HORM4_610131**	47,9	vv2_0094	TadF
**HORM4_610132**	48,4	vv2_0095	TadG
**HORM4_430096**	48,1	vv1AM_1569	Flp-a

**HORM4_430098**	34,1	vv1_1746	TadV
**HORM4_430099**	62,4	vv1_1747	ATP-ase
**HORM4_430100**	61,5	vv1_1748	RcpC
**HORM4_430101**	70,8	vv1_1749	RcpA
**HORM4_430102**	49,3	vv1_1750	RcpB
**HORM4_430103**	53,8	vv1_1754	TadG
**HORM4_430104**	75,6	vv1_1755	TadA
**HORM4_430105**	57,6	vv1_1756	TadB
**HORM4_430107**	55,8	vv1_1757	TadC
**HORM4_430108**	55,2	vv1_1758	TadD

Table S3. *V. harveyi* ORM4 CPS proteins and amino acidic percentage of identity with CPS proteins of *V. parahaemolyticus* RIMD2210633.

Vibrio harveyi **ORM4 gene ID**	**% aa identity**	***Vibrio parahaemolyticus*** **RIMD2210633 gene ID**	***Vibrio parahaemolyticus*** **RIMD2210633 protein**
**HORM4_940043**	86.27	vpa1403	CpsA
**HORM4_940044**	71.32	vpa1404	CpsB
**HORM4_940045**	89.83	vpa1405	CpsC
**HORM4_940046**	85.53	vpa1406	CpsD
**HORM4_940047**	71	vpa1407	CpsE
**HORM4_940048**	78.57	vpa1408	CpsF
**HORM4_940049**	73.12	vpa1409	CpsG
**HORM4_940050**	86.96	vpa1410	CpsH
**HORM4_940052**	76.38	vpa1411	CpsI
**HORM4_940053**	67.36	vpa1412	CpsJ
**HORM4_940054**	72.8	vpa1413	CpsK

Table S4. Expression of genes associated to the *cps* cluster in *V. harveyi* ORM4 biofilm cells.

Vibrio harveyi **ORM4 gene ID**	**FC**	**Log_2FC**	**Adjusted *p*-value**
**HORM4_940043**	2.192	1.132	0.05
**HORM4_940044**	1.503	0.588	0.3
**HORM4_940045**	1.463	0.549	0.32
**HORM4_940046**	1.33	0.412	0.51
**HORM4_940047**	0.946	-0.08	0.89
**HORM4_940048**	1.198	0.26	0.62
**HORM4_940049**	1.107	0.147	0.8
**HORM4_940050**	1.486	0.572	0.22
**HORM4_940052**	1.382	0.467	0.4
**HORM4_940053**	1.431	0.517	0.30
**HORM4_940054**	1.636	0.71	0.15
